# Analyzing correlation between epicardial fat area and metabolic syndrome risk factor by using low-dose Lung CT

**DOI:** 10.12669/pjms.315.7991

**Published:** 2015

**Authors:** Hyon-Chol Jang, Hae-Kag Lee, Heon Lee, Jang-Gyu Cha, Yoon-Shin Kim, Jae-Hwan Cho

**Affiliations:** 1 Hyon-Chol Jang, PhD. Department of Radiological Science, Suseong College, Daegu 706-711, Republic of Korea. Department of Health Science, Hanyang University, Seoul 133-791, Republic of Korea; 2 Hae-Kag Lee, PhD. Department of Computer Science and Engineering, Soonchunhyang University, Asan, Republic of Korea; 3 Heon Lee, PhD. Department of Radiology, Soonchunhyang University Hospital, Bucheon 420-767, Republic of Korea; 4 Jang-Gyu Cha, PhD. Department of Radiology, Soonchunhyang University Hospital, Bucheon 420-767, Republic of Korea; 5 Yoon-Shin Kim, PhD. Department of Health Science, Hanyang University, Seoul 133-791, Republic of Korea; 6 Jae-Hwan Cho, PhD. Department of International Radiological Science, Hallym University of Graduate Studies, Seoul, Republic of Korea

**Keywords:** Blood Count, Epicardial Area, Metabolic Syndrome, Metabolic Risk Factors

## Abstract

**Objectives:**

To study about the blood count of a risk factor related to physical measurement and metabolic syndrome, and the area of epicardial fat for medical checkup patients.

**Methods:**

From April 1^st^ to November 15^th^ in 2014, we measured the area of epicardial fat in the adult out patients under 60 years of age, who are in good health; and the patients took the blood test and low-dose lung CT. In order to identify the relationship between the area of epicardial fat and the risk factor of metabolic syndrome, we conducted correlation analysis. Then, we performed multiple regression analysis to evaluate an independent correlation of epicardial area. In addition, we computed the cut-off value of epicardial fat area by using ROC (Receiver Operating Characteristic) curve to foresee a metabolic syndrome factor that has the most proper sensitivity and specificity.

**Results:**

Waist circumference, fasting blood sugar, triglyceride, high-density lipoprotein (HDL) cholesterol, systolic blood pressure, and diastolic blood pressure were shown to be the factors that affect the area of epicardial fat. Therefore, if waist circumference, fasting blood sugar, triglyceride, systolic blood pressure, and diastolic blood pressure were increased, the area of epicardial fat would be significantly increased (P<0.05); and if high-density lipoprotein cholesterol was increased, the area of epicardial fat would be significantly decreased (P<0.05). Out of metabolic syndrome factors, waist circumference’s ROC curve area was 0.79 (Confidence Interval 0.73-0.84, P<0.05), which was the highest. The sensitivity was 83.7% when specificity was 70.1%, which proves that they are important factors for the diagnosis. In brief, metabolic syndrome is a disease that mostly appears in obesity patients, so we should try to monitor and cure the disease.

**Conclusion:**

The risk factors of metabolic syndrome can be managed through health care, and if we try to decrease the risk factors, we will be able to shrink epicardial fat area and decrease metabolic syndrome at the same time.

## INTRODUCTION

The main factor that causes the metabolic disease is obesity by intake of instant food products or foods with high calories and high fat, increased stress level, and lack of exercise.^1^,^2^

Obesity is a condition where fat has largely/abnormally accumulated in the body, threatening the health condition; this condition is widespread all over the world as a chronic disease.^3^,^4^ Furthermore, the outbreak of cardiovascular disease and metabolic disease due to obesity is constantly increasing, regardless of gender, age, and race.^5^,^6^

The growth of obesity also shortens people’s life span and encourages the outbreak of metabolic disease.^7^,^8^ Recently, epicardial fat is being studied as a new indicator that predicts any internal fat within the thorax.^9^-^11^ Epicardial fat is embryologically the same as intra-abdominal fat,^12^ so if we take a closer look at it, we may be able to prevent or cure the outbreak of cardiovascular and metabolic diseases by predicting the diseases in advance. However, we need technological skills for measuring epicardial fat, because the entire result can become a failure even with a slight miscalculation. Moreover, the measurement may not be precise, depending on the measurement methods.

In the case of using a cardiac ultrasound, the measurement may not be precise due to the unclear border zone and the various distribution of epicardial fat according to its location. In the case of using a cardiac MRI, it is impossible to use an artificial pacemaker or clip used for blood vessel treatment in the patients who have claustrophobia, and it also takes a long time and costs a lot of money. In the case of using a cardiac CT, there can be side effects from the possibility of being highly exposed to radiation, due to the usage of contrast media; so, we need to work on that as well.^13^,^14^ However, even though there are many clinical studies conducted with escalated attention on the epicardial fat, they are only depending on cardiac ultrasound, cardiac MRI, and cardiac CT. Therefore, this study aimed to find the correlation between metabolic syndrome risk factors, by using low-dose lung CT which is a not invasive and is low in radiation exposure. So far, there is only a few studies reporting on epicardial fat area and metabolic syndrome risk factor measured by low-dose lung CT.

Generally, low-dose lung CT is used for an early diagnosis of lung cancer and solitary pulmonary nodule. It is low on radiation exposure, takes a short time, and has no side effects regarding the contrast media.^15^,^16^ Therefore, we conducted this study to confirm the correlation by identifying the relationship between metabolic syndrome risk factor and epicardial fat area measured by low-dose lung CT.

## METHODS

We randomly chose 194 healthy adults with less than 60 years of age, among the medical check-up patients who underwent low-dose lung CT and blood test together, from April 1^st^ to November 15^th^ in 2014. The physical characteristics of the patients are shown in Table-I.

**Table-I:** Subject characteristics (n = 194).

	Minimum	Maximum	Mean	SD
Age (years)	23	60	43.66	7.70
WC (cm)	59	103	82.91	7.92
FBS (mg/dL)	67	264	95.13	20.47
TG (mg/dL)	31	534	149.12	94.24
HDL-C (mg/dL)	27	94	51.19	11.45
SBP (mmHg)	87	172	122.52	14.63
DBP (mmHg)	49	104	75.36	10.43

* WC, Waist Circumference; FBS, Fasting blood sugar; TG, Triglyceride; HDL-C, High density lipoprotein cholesterol; SBP, Systolic Blood pressure; DBP, Diastolic Blood pressure.

### Method

**Fig.1:**
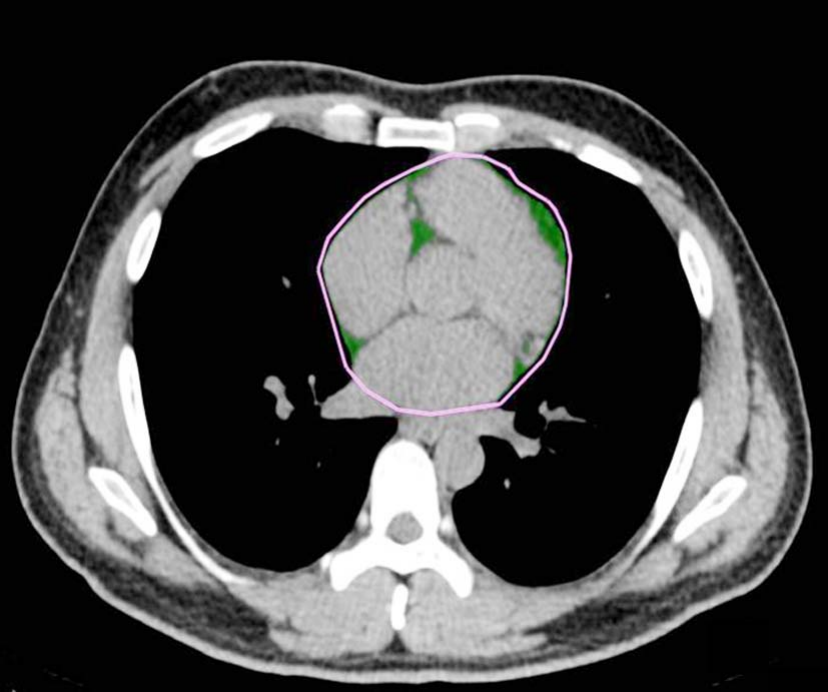
Epicardial fat area measurement. The Epicardial fat area (green color).

In order to measure epicardial fat area, we used 64-MDCT (LightSpeed VCT Xte, GE Healthcare, Milwaukee, USA); and to obtain an image by low-dose lung CT, we set the tube voltage to 120kVp, tube current to 15mA, scan type to axial, rotation time to 0.5sec, detector coverage to 40mm, DFOV to 35cm, and Matrix size to 51. We covered the entire lung, from lung apex to costopherenic angle, when the subject inhaled. By using an image reproduce workstation, advantage workstation (version 4.4, GE, USA), we reproduced and obtained the image to a 3mm of slice. We measured and analyzed the epicardial fat area with the two skilled cardio radiologists, by using Aquarius workstation (Terarecon, Inc, San Mateo, CA, USA) which is made to measure the epicardial fat area. From the image that shows the first part of the heart and to the one that shows the lowest part of the heart, we chose the middle part to measure the epicardial fat area. In order to measure it, we drew the region of interest (ROI) in an epicardial fat area with the green color, so that we could measure only the part marked with green. We measured the epicardial fat area with the value computed by program analysis that uses CT value’s attenuation scope between -200HU ~ -30HU (Hounsfield unit, HU) (Fig.1). For the physical measurements, we measured the patients’ heights and weights using an automatic scale. For measuring waist circumference, a skilled expert measured the middle part that is between the lower part of costal bone and iliac crest. In order to check the blood pressure, we let the subject to relax in a sitting position for about 10 minutes, and then measurement was done by using a hydrargyrum blood pressure manometer. We selected metabolic syndrome risk factors based on AHA/NHLBI^17^ in 2005, and we chose waist circumference for Asians, based on world health organization (WHO) of western pacific region.^18^ We divided metabolic syndrome risk factors into 6 categories as follows: waist circumference (normal scale: less than 90cm in men, less than 80cm in women), fasting blood sugar (normal scale: less than 100mg/dL), triglyceride (normal scale: 150mg/dL), high density lipoprotein cholesterol (normal scale: over 40mg/dL in men, over 50mg/dL in women), systolic blood pressure (normal scale: less than 130mmHg), and diastolic blood pressure (normal scale: less than 85mmHg).

### Statistical analysis method

We recorded the technical data in average±standard deviation and percentage. In order to identify the differences between epicardial fat area and metabolic syndrome risk factors, we performed the independent sample, T-test, and conducted confidence test between evaluators for the measurement of the epicardial fat area. In addition, we performed a correlation analysis to find the relationship between epicardial fat area and risk factors, and then conducted a multiple regression analysis to assess the independent correlation with the epicardial fat area. Moreover, we computed the cut-off value of epicardial fat area by using ROI curve, to anticipate the metabolic syndrome risk factor with the most proper sensitivity and specificity. We conducted a statistic analysis by using SPSS ver. 18.0 software, and the significant level was set to less than 0.05.

## RESULTS

### Characteristic of epicardial fat area

Since two evaluators measured the epicardial area, we conducted the confidence test. As a result of computing intra-class correlation coefficient (ICC), the conformity degree was quite high, showing 0.997 (P<0.05). The average value of epicardial fat area was 11.79±5.65cm^2^, and the average CT value was -89.45±3.72 HU.

### Analyzing the differences in epicardial fat area depending on metabolic syndrome factors

The differences in epicardial fat area depending on metabolic syndrome factors are shown in Table-II. All of the factors for metabolic syndrome showed a significant difference (P<0.05). For waist circumference, the obesity group showed a larger area of epicardial fat than the normal group, by showing 16.84±7.05cm^2^ (P<0.05). Also, for the fasting blood sugar, triglyceride, high density lipoprotein cholesterol, systolic blood pressure, and diastolic blood pressure, the obesity group showed a larger area of epicardial fat than the normal group, by showing 15.49±8.22cm^2^, 14.51±7.63cm^2^, 10.88±5.52cm^2^, 12.98±5.70cm^2^, and 13.17±5.6cm^2^ (P<0.05), respectively.

**Table-II:** Analysis on the differences between Epicardial fat areas depending on metabolic syndrome factors.

Epicardial Fat area (cm^2^)					
Factor	Normal		Obesity		
	Mean	SD	Mean	SD	P
WC (cm)	10.22	4.04	16.84	7.05	0.00

Factors	Normal		Abnormal		
	Mean	SD	Mean	SD	P
FBS (mg/dL)	11.29	5.05	15.49	8.22	0.00
TG (mg/dL)	11.17	4.92	14.51	7.63	0.00
HDL-C (mg/dL)	12.75	5.67	10.88	5.52	0.02
SBP (mmHg)	10.22	5.23	12.98	5.70	0.00
DBP (mmHg)	11.04	5.52	13.17	5.67	0.01

* WC, Waist Circumference; FBS, Fasting blood sugar; TG, Triglyceride; HDL-C, High density lipoprotein cholesterol; SBP, Systolic Blood pressure; DBP, Diastolic Blood pressure.

### Correlation between metabolic syndrome factors and epicardial fat area.

As a result of identifying the correlation between metabolic syndrome factors and epicardial fat area, as shown in Table-III, the factors such as waist circumference, fasting blood sugar, triglyceride, high density lipoprotein cholesterol, systolic blood pressure, and diastolic blood pressure turned out to be correlated with epicardial fat area (P<0.05).

**Table-III:** Correlation analysis between epicardial fat area and metabolic syndrome factors.

	Epicardial Fat area (cm^2^)
WC (cm)	0.61^†^
FBS (mg/dL)	0.17*
TG (mg/dL)	0.37^†^
HDL-C (mg/dL)	-0.27^†^
SBP (mmHg)	0.24^†^
DBP (mmHg)	0.22^†^

* WC, Waist Circumference; FBS, Fasting blood sugar; TG, Triglyceride;

HDL-C, High density lipoprotein cholesterol;

SBP, Systolic Blood pressure;

DBP, Diastolic Blood pressure.

* P < 0.05,

† P < 0.01

### Analyzing metabolic syndrome factors that affect epicardial fat area

Table-IV shows the result of multiple regression analysis identifying the metabolic syndrome factors that affect epicardial fat area. The factors affecting epicardial fat area are waist circumference, fasting blood sugar, triglyceride, high-density lipoprotein cholesterol, systolic blood pressure, and diastolic blood pressure. Therefore, when waist circumference, fasting blood sugar, triglyceride, systolic blood pressure, and diastolic blood pressure rise, epicardial fat area becomes significantly larger (P<0.05). Also, when high-density lipoprotein cholesterol increases, epicardial fat area becomes significantly smaller (P<0.05).

**Table-IV:** Multiple regression analysis between epicardial fat area and metabolic syndrome factors.

Factors	β	SE	R2	P
WC (cm)	0.43	0.41	0.374	0.00
FBS (mg/dL)	0.04	0.02	0.029	0.02
TG (mg/dL)	0.02	0.01	0.142	0.00
HDL-C (mg/dL)	-0.13	0.03	0.074	0.00
SBP (mmHg)	0.09	0.02	0.055	0.00
DBP (mmHg)	0.12	0.03	0.050	0.00

* WC, Waist Circumference; FBS, Fasting blood sugar; TG, Triglyceride; HDL-C, High density lipoprotein cholesterol; SBP, Systolic Blood pressure; DBP, Diastolic Blood pressure.

### Analyzing predictive metabolic syndrome factors that affect epicardial fat area

**Fig.2:**
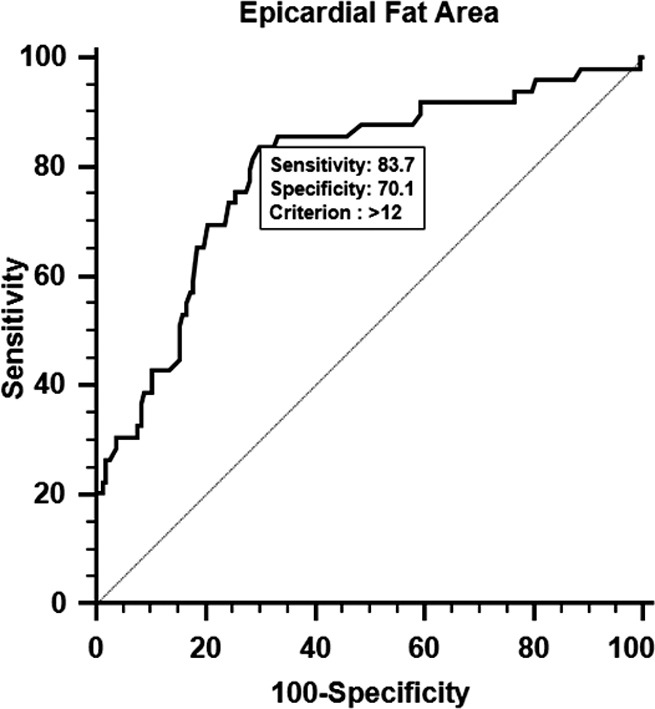
Analysis on ROC curve of epicardial fat area for WC (Waist Circumference)

Table-V shows the result of ROC curve analysis for the predictive metabolic syndrome factors that affect epicardial fat area. Out of metabolic syndrome factors, waist circumference’s ROC curve area was shown to be the highest, by showing the confidence interval of 0.73-0.84 (P<0.05). This appeared as an important factor to analyze, by representing 83.7% of sensitivity and 70.1% of specificity (Fig.2).

**Table-V:** Analysis on predictive metabolic syndrome factors affecting epicardial fat area.

Factors	AUC	95% CI	Sensitivity (%)	Specificity (%)
WC (cm)	0.79	0.73-0.84	83.7	70.1
FBS (mg/dL)	0.65	0.58-0.71	37.5	91.8
TG (mg/dL)	0.63	0.56-0.70	55.3	73.2
HDL-C (mg/dL)	0.60	0.53-0.67	55.2	65.3
SBP (mmHg)	0.66	0.59-0.72	60.9	69.2
DBP (mmHg)	0.63	0.56-0.69	65.3	63.4

* WC, Waist Circumference; FBS, Fasting blood sugar; TG, Triglyceride; HDL-C, High density lipoprotein cholesterol; SBP, Systolic Blood pressure; DBP, Diastolic Blood pressure.

## DISCUSSION

Epicardial fat area is known to be relevant to metabolic syndrome and cardiovascular disease, as well as to the growth of prevalence rate of metabolic syndrome disease.^19^,^20^ Since people these days are more concerned about epicardial fat, various studies are being conducted. Most of them are conducted by using heart MRI, heart CT, and heart ultrasound for measuring the thickness and volume of fat. Because epicardial fat is largely distributed, depending on the location, it is hard to clearly section the areas. Furthermore, whenever we use a different method, it is hard to obtain a precise value. Therefore, we should let a skilled expert to measure the epicardial fat area for a precise measurement.^14^ For the heart MRI, it is impossible to proceed with the test when there is a pacemaker or clip equipment used for vessel treatment, or when the subject has claustrophobia. Furthermore, MRI test is not efficient because it is expensive and it also takes a long time.^21^ When we conduct heart CT test, we are likely to be exposed to radiation, so we need to work on reducing the radiation exposure. Also, we need to be careful about any side effects due to the use of contrast media, because this test also requires contrast media, similar to other CT tests.^22^,^23^

This study is meaningful because we found that there is a correlation between epicardial fat area and metabolic syndrome factors, by using low-dose lung CT that is not likely to cause any side effects when using contrast media. For the tests used in this study, it is less likely to be exposed to radiation, and it only takes a short amount of time. An image that we obtained through low-dose lung CT test may not portray a clear boundary of pericardium due to the heartbeat. Therefore, heart CT that uses ECG-gated can improve the image quality, and it could produce more precise results than this study. However, this study proved its conformity on coronary artery calcification score through the comparative study of non-ECG gated CT test and ECG-gated CT test.^24^ This is why we believe that more meaningful results can be obtained if we do more study with ECG-gated, since we have only tested with non-ECG gated. In addition, we will be able to improve the image quality if the radiologist train the subjects to have them practice breathing before undergoing the low-dose lung CT.

As a result of computing intra-class correlation coefficient (ICC), the conformity of the two evaluators was quite high, by showing 0.997 (P<0.05). Therefore, we were able to confirm the confidence level of epicardial fat area measured by low-dose lung CT. As a result of performing a multiple regression analysis, waist circumference, fasting blood sugar, triglyceride, high-density lipoprotein cholesterol, systolic blood pressure, and diastolic blood pressure turned out to affect the area of epicardial fat. Therefore, we found that when waist circumference, fasting blood sugar, triglyceride, systolic blood pressure, and diastolic blood pressure increase, the area of epicardial fat becomes larger (P<0.05); and when high density lipoprotein cholesterol increases, the area of epicardial fat significantly becomes smaller (P<0.05). Kim and others^25^ reported that when metabolic syndrome risk factors are increased, the area of epicardial fat becomes significantly increased (P<0.05). As a result of analyzing ROC curve, for identifying predictive metabolic syndrome factors that affect epicardial fat area, waist circumference turned out to be an important factor for the analysis, by showing 83.7% of sensitivity and 70.1% of specificity. When metabolic risk factors such as waist circumference, fasting blood sugar, triglyceride, high-density lipoprotein cholesterol, systolic blood pressure, and diastolic blood pressure belong to an abnormal group, epicardial fat area becomes larger. These metabolic syndrome risk factors could be managed through a good health care; and if we reduce epicardial fat area, it will also reduce the risk for metabolic diseases.

## CONCLUSIONS

Since most of metabolic diseases are found in obesity patients, we should try to prevent and treat these diseases. We can manage metabolic syndrome risk factors through health care, by staying in shape. Therefore, by reducing those factors, we not can only reduce epicardial fat area but also reduce the risks for metabolic diseases. We will need a further prospective study on the subjects with metabolic disease.
